# Dual phenotypic characteristics of P-selectin in a mouse model of hemorrhagic shock and hepatectomy

**DOI:** 10.1016/j.heliyon.2023.e18627

**Published:** 2023-07-28

**Authors:** Jen-Lung Chen, Tzu-Ting Cheng, Chien-Chi Huang, Hsin-Hou Chang, Chen-Fuh Lam

**Affiliations:** a Division of General Surgery, Department of Surgery, E-Da Hospital, I-Shou University, Kaohsiung, 824, Taiwan; b Department of Anesthesiology, E-Da Hospital, I-Shou University, Kaohsiung, 824, Taiwan; c Department of Medical Research, E-Da Hospital, I-Shou University, Kaohsiung, 824, Taiwan; d Department of Molecular Biology and Human Genetics, Tzu-Chi University, Hualien, 907, Taiwan; e Department of Anesthesiology, Dalin Tzu Chi Hospital, Buddhist Tzu Chi Medical Foundation, Chia-Yi, 622, Taiwan

**Keywords:** Trauma, Hemorrhagic shock, Liver regeneration, P-selectin, Inflammation

## Abstract

**Background:**

Membrane-bound P-selectin induces endothelial adhesion of leucocytes and amplifies organ inflammations during major trauma, while soluble P-selectin (sP-sel) mediates survival rescue properties. This study characterized the differential effects of P-selectin in a “2-hit” model of hemorrhagic shock (HS) and partial hepatectomy (PH).

**Materials and methods:**

HS was induced by withdrawing blood (0.3 mL) directly from the mouse femoral arteries. 70% or 50% of liver volumes were resected after inducing HS. Time of survival in P-selectin deficient (Selp −/−) mice treated with and without intraperitoneal injections of recombinant P-sel IgG-Fc fusion proteins (rP-sel-Fc, 1.5 mg/kg) were recorded for up to 72h after injury. In addition, liver regeneration at 72h after HS and 50% PH was assessed in wild-type and Selp −/− mice.

**Results:**

Compared to wild-types, Selp −/− mice had significantly higher mortality rates post HS and 70% PH, as none of these animals survived up to 48h postoperatively. The survival curve was restored in Selp −/− mice pre-treated with rP-sel-Fc. In the HS followed by 50% PH experimental arm, liver remnant growth ratios were significantly higher in Selp −/− mice (15.7 ± 3.1 vs 11.7 ± 4.9, P = 0.040). The elevated serum concentrations of alanine aminotransferase post-PH were significantly reduced in Selp −/− mice. Hepatocyte proliferation indices (CYP7a1 and PCNA) expression were enhanced and myeloperoxidase activity in the regenerated remnant liver was reduced in the Selp −/− mice.

**Conclusion:**

In critical conditions induced by HS and PH, P-selectin mediates two distinct phenotypic characteristics. Soluble-form circulating P-selectin improves survival in the acute stage of HS and extensive loss of liver parenchyma; membrane-bound P-selectin induces regional pro-inflammatory reactions in the remnant liver after the acute stage of two insults, thereby inhibiting hepatic regeneration. The results of this pre-clinical study may provide molecular mechanistic insight and clinical therapeutic applications of P-selectin in the acute and regenerative phases of traumatic hepatic injury.

## 1 Introduction

The anterior location of the liver in the abdomen and its fragile parenchyma makes the liver very susceptible to damage and rupture during blunt abdominal trauma or direct penetrating injuries. Liver laceration, or hepatic rupture, is a life-threatening event that usually requires an urgent laparotomy for hemostasis or partial hepatectomy to remove the damaged liver tissue. Approximately 5% of all trauma cases admitted to the emergency department are associated with liver injuries, and 10–15% of these patients who pass away shortly after abdominal trauma have high grade liver injuries [1]. In unstable patients with high grade hepatic trauma (American Association for Surgery of Trauma (AAST) grade III-V), surgical resection of the damaged liver parenchyma is a standard life-saving treatment [[2], [3], [4]]. However, extremely high mortality rates (30–68%) are still reported in patients with high grade hepatic trauma even after urgent hepatectomies due to hepatic or multiple organ failure [5].

In liver injuries, activated Kupffer cells trigger a proinflammatory cascade leading to the recruitment of leukocytes, which aggravates parenchymal injury [6]. P-selectin is an important adhesion molecule expressed on the endothelial lumen, which is responsible for the initial rolling and attachment of activated circulating inflammatory cells and platelets during acute inflammatory processes [7]. The increased infiltration of neutrophils directly injure hepatocytes via the release of oxidants and proteases, leading to necrotic cell death and tissue damage [6]. In an experimental model of hepatic warm ischemia-reperfusion injury, the survival rate at 90 min after injury was significantly higher in mice with P-selectin deficiency, as neutrophil adhesion and platelet sequestration was significantly reduced in these animals [8]. During hemorrhagic shock, P-selectin activation in the microvascular endothelium is essential for the initial upregulation of the systemic inflammatory response [9]. P-selectin neutralization in the early phases of hemorrhagic shock has been shown to reduce leukocyte infiltration in the inflamed liver [9,10] and help maintain a significantly higher blood pressure following resuscitation [9]. However, the effects of P-selectin on survival outcomes during the acute resuscitation period and liver regeneration in later recovery phases after hemorrhagic shock and partial hepatectomy are undetermined. This study hypothesized that genetical deletion of P-selectin might reduce inflammatory cell infiltration and attenuate pro-inflammatory responses in the remnant liver, thereby improving the overall mortality rate and liver regeneration in mouse models of hemorrhagic shock and partial hepatectomy.

## 2 Materials and Methods

### 2.1 2-1. Mouse models of hemorrhagic shock resuscitation (HSR) and partial hepatectomy (PH)

The animal studies were conducted in compliance with the guidelines set by the E-Da Cancer Hospital Animal Center and approved by the Institution of Animal Care and Use Committee (Approval #EDCH 106006, E-Da Cancer Hospital, Kaoshiung, Taiwan). C57BL/6 wild-type mice or C57BL/6 mice deficient in P-selectin (Selp−/−) (weighing approximately 20 g) were anesthetized using an intraperitoneal injection of cocktail anesthetics (Zoletil, 50 mg/kg). The femoral arteries of the mice were cannulated with a 30G polyethylene catheter (PE10, I.D. 0.28 mm) and connected to a pressure transducing system (Kent Scientific Corporation, Torrington, CT, USA) for blood pressure monitoring and aspiration of blood samples. A total of 300 μL blood was slowly taken from a 3-way stopcock in the transducing system over 10 min while maintaining a low mean arterial pressure of approximately 40 mmHg for 30 min (hypovolemic shock phase). [11] An equal amount of normal saline to the amount of blood taken was slowly infused through the arterial cannula to restore the circulatory volume (resuscitation phase).

Ten minutes after resuscitation, the upper abdomen and lower lateral portions of both hemi-thoraces were compressed to exteriorize the liver following midline laparotomy. The liver lobes were visualized and mobilized to identify the ligaments of the right and left lobes. The stems of the liver lobes to be resected were sutured and ligated using 5-0 nylon sutures. The ligated liver lobes were then carefully resected above the knot with a microdissecting scissors. For a 70% PH, the right medial, left medial and left lateral lobes were excised en bloc [12]. For a 50% PH, the left medial and left lateral lobes were excised en bloc [12]. The remaining remnant liver lobes were replaced into the abdominal cavity and the abdominal wall was then closed in layers. Animals were allowed to recover spontaneously from anesthesia in a heated incubator and they had free access to food and water after surgery.

### 2.2 2-2. Animals and treatment protocols

C57BL/6 wild-type mice were purchased from the BioLASCO Corp. (Taipei, Taiwan) and Selp−/− mice were obtained from the laboratory of Professor Hsin-Hao Chang. The Selp−/− mice were back-crossed with the parental C57BL/6 mouse strain for at least six generations. In the survival study, Selp−/− mice were randomly assigned to receive either an intraperitoneal injection of recombinant P-sel IgG-Fc fusion protein (rP-sel-Fc; 1.5 mg/kg, R&D Systems, MN) or normal saline at 30 min before 70% PH; while the wild-type mice only underwent the 70% PH. Signs of moribund or mortality were closely observed for up to 72h post-operatively. Remnant liver tissues were harvested from mice receiving HSR and 70% PH that survived more than 72h for analysis. In the other quantitative studies, wild-type and Selp−/− mice were randomly assigned to receive 50% PH with or without HSR. The animals were asphyxiated with CO2 at 3 days post-operatively and blood samples as well as the regenerated remnant livers were harvested for analysis.

### 2.3 2-3. Analysis of blood biochemistries and tissue cytokines

Serum titers of liver function, including alanine aminotransferase (ALT), albumin, and total bilirubin were analyzed by an autoanalyzer (Quik-Lab). Tissue concentrations of inflammatory cytokines (monocyte chemoattractant protein (MCP)-1, interleukin (IL)-6, and tumor necrosis factor (TNF)-α) in the remnant liver were determined using the cytokine ELISA assay kits (Signosis Inc.) according to the manufacturer's instructions.

### 2.4 2-4. Liver mass and growth ratio

Liver masses were determined immediately after excision. The initial remnant liver mass (right liver lobe ≈50% of the total liver mass, grams) was estimated as 50% of the tissue excised. Liver mass was normalized by the body weight measured before and after hepatectomy (liver-to-body mass ratio). Liver growth ratio was computed by the division of preoperative liver mass by the post-hepatectomy liver mass.

### 2.5 2-5. Tissue myeloperoxidase (MPO) activity and 3-nitrotyrosine

The enzymatic activity of myeloperoxidase (MPO) in the homogenized liver was measured by a commercially available MPO assay kit (Cell Biolabs Inc., San Diego, CA). An ELISA kit was used to determine the levels of 3-nitrotyrosine in the liver tissues (FineTest, Wuhan, China).

### 2.6 2-6. Western blot

Soluble protein extracts of liver tissue were loaded into polyacrylamide gels and transferred onto nitrocellulose membranes. Anti-vascular endothelial growth factor receptor (VEGFR)-2 (rabbit polyclonal, 1:500 dilution; NOVUS Biologicals; Centennial, CO), hepatocyte growth factor (HGF) (rabbit polyclonal, 1:1000 dilution; GeneTex; Irvine, CA), cholesterol-7-alpha-dydroxylase (CYP7a1) (rabbit polyclonal, 1:1000 dilution; Abcam; Cambridge, UK) and proliferating cell nuclear antigen (PCNA) (mouse monoclonal,1:1000 dilution; BD Biosciences; Franklin Lakes, NJ) antibodies were used. After incubation with HRP-linked secondary antibodies, bands were visualized by enhanced chemiluminescence and quantified using the ImageJ (1.48v, NIH).

### 2.7 2-7. Histological examination and immunohistochemical staining

Liver tissues were immersed in 4% formaldehyde for 24 h and the paraffin-embedded biopsies were sectioned. Tissues were processed for Hematoxylin and Eosin (HE) stain and analyzed under microscopy. Paraffin-embedded tissues were sectioned for immunohistochemical staining processing. Mouse monoclonal *anti*-PCNA (1:100) and Ki67 (1:100) antibodies (BD Biosciences; Franklin Lakes, NJ) were used to visualize the expressions on the liver sections using the Avidin-Biotin Complex method (Vector Laboratories).

### 2.8 2-8. Statistical analysis

Differences in survival rates were analyzed using the log-rank tests for Kaplan-Meier curves. Values of continuous variables in different experimental groups were compared using the Kruskal-Wallis test by rank or Wilcoxon Rank Sum Test. Results are presented as the median and interquartile range (IQR). Statistical significance was accepted at a level of *P* < 0.05. All statistical analyses were performed using the SigmaPlot 14.0 (Systat Software Inc., San Jose, CA).

## 3 Results

### 3.1 3-1. Pretreatment with soluble P-selectin (rP-sel-Fc) improved survival outcomes after 2-hit injury of HSR and 70% PH

**Fig. 1:**
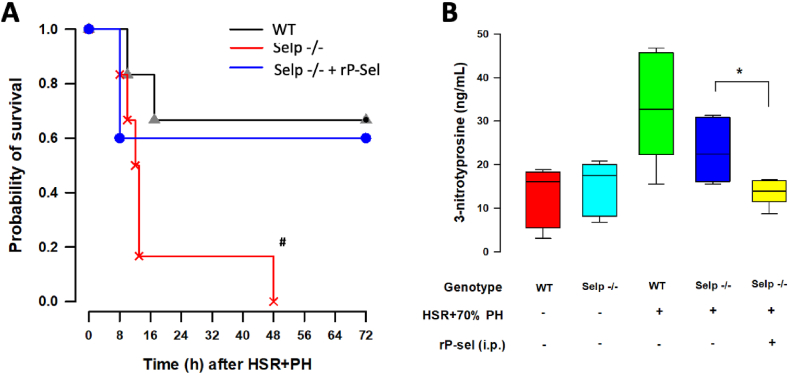
**(A).** Kaplan-Meier plots for estimating the overall survival rates of wild-type (WT) and P-selectin deficient (Selp −/−) mice up to 72h after induced hemorrhagic shock (HS) and 70% partial hepatectomy (PH). In some Selp −/− mice, recombinant P-sel IgG-Fc fusion protein (rP-sel-Fc, 1.5 mg/kg) was administered intraperitoneally before HS to increase circulating levels of soluble P-selectin. Time-to-event analysis showed that none of the Selp −/− mice survived up to 48h (^#^P = 0.018 vs WT), but the survival rate was restored in Selp−/− mice that were pre-treated with rP-sel-Fc. Survival curves were compared with log-rank test, n = 5–6 different animals in each group. **(B).** Remnant liver tissues were harvested from mice receiving HS and 70% PH that survived more than 72h for analysis of tissue concentrations of 3-nitrotyrosine (3-NT). Enhanced 3-NT in the remnant livers of Selp−/− mice were significantly suppressed by intraperitoneal injection of rP-sel-Fc. *P = 0.013, n = 5–6 different animals in each group; data were analyzed using the Kruskal-Wallis test by rank. Results are presented as box-and-whisker plots, in which the horizontal lines of color boxes indicate the 75th percentile, median and 25th percentile of the distribution, and the upper and lower whiskers indicate the maximal and minimal values. (For interpretation of the references to color in this figure legend, the reader is referred to the Web version of this article.)

Compared with wild-type mice, Selp−/− animals were found to have significantly higher mortality rates at 8h after HSR and 70% PH, and none of the P-selectin deficient mice survived up to 48h (Fig. 1A). However, the survival rate was restored in Selp−/− mice that were pre-treated with rP-sel-Fc (Fig. 1A). Tissue concentrations of 3-nitrotyrosine in the remnant livers of Selp−/− mice were also significantly suppressed by intraperitoneal rP-sel-Fc administration (Fig. 1B).

### 3.2 3-2. General outcomes of 2-hit injury of HSR and 50% PH

**Fig. 2:**
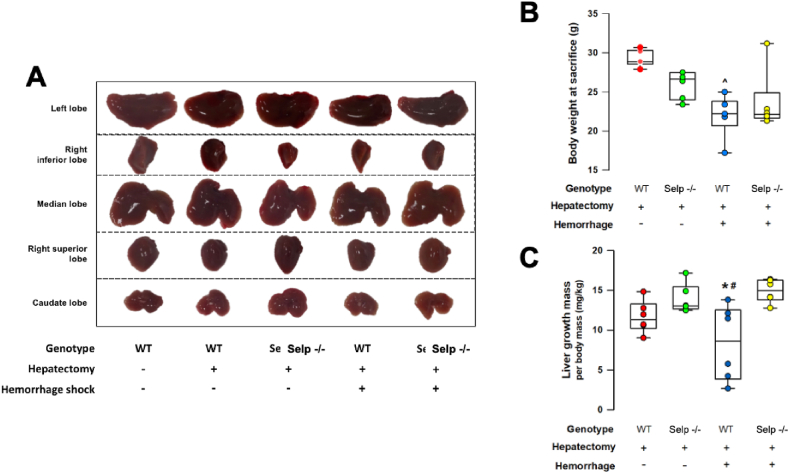
Clinical outcomes at 72h after induced hemorrhagic shock (HS) and 50% partial hepatectomy (PH). **(A).** Gross autopsies of the regenerated remnant liver lobes at 72h after HS and 50% PH in wild-type (WT) and P-selectin deficient (Selp −/−) mice. **(B).** Physiological stress in experimental mice was determined by changes in body weight at the time of sacrifice. Body weight of wild-type animals that received the 2-hit injury was significantly lower than wild-type animals that received only PH (^P = 0.028 vs WT with PH only). **(C).** The regeneration of remnant liver lobes was assessed by the liver growth ratio and was normalized by the body mass at the time of sacrifice. The liver growth ratio was significantly reduced in WT mice received HS and 50% PH in comparison to PH only groups and Selp −/− mice. *P = 0.010 vs Selp −/− with HS + PH; ^#^P = 0.026 vs Selp −/− with PH only. n = 6 different animals in each group; data were analyzed using the Kruskal-Wallis test by rank. Results are presented as box-and-whisker plots, in which the horizontal lines of color boxes indicate the 75th percentile, median and 25th percentile of the distribution, and the upper and lower whiskers indicate the maximal and minimal values. (For interpretation of the references to color in this figure legend, the reader is referred to the Web version of this article.)

No surgical-related mortality up to 72h after HSR and 50% PH was recorded in wild-type or Selp−/− mice. At the time of sacrifice, the body weight of wild-type animals that received the 2-hit injury was significantly lower than the animals that only received 50% PH, suggesting that hemorrhagic shock precipitates additional physiological stress after PH (Fig. 2B). The average postoperative body weights were similar in Selp−/− mice that underwent the 2-hit injury and those that only underwent 50% PH (Fig. 2B).

### 3.3 3-3. Improved liver growth mass and liver function in P-selectin deficiency mice after 2-hit injury of HSR and 50% PH

**Fig. 3:**
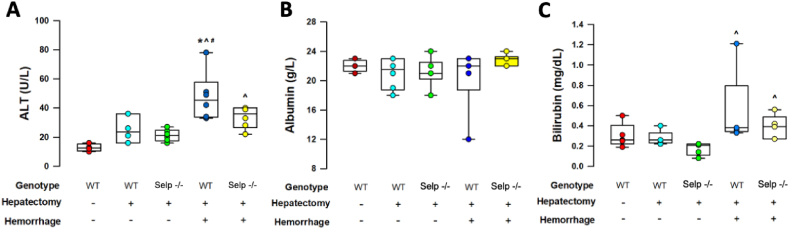
Liver function at 72h after 50% partial hepatectomy (PH) with or without hemorrhagic shock (HS) was assessed by analysis of serum concentrations of alanine aminotransferase ALT (liver enzyme), total bilirubin (liver metabolism), and albumin (liver biosynthesis) in wild type (WT) and P-selectin deficient (Selp −/−) mice. *P = 0.002 vs Selp −/− with HS + PH; ^P < 0.05 vs the PH only within groups; ^#^P = 0.020 vs Selp −/− with PH only. n = 6 different animals in each group; data were analyzed using the Kruskal-Wallis test by rank. Results are presented as box-and-whisker plots, in which the horizontal lines of color boxes indicate the 75th percentile, median and 25th percentile of the distribution, and the upper and lower whiskers indicate the maximal and minimal values. (For interpretation of the references to color in this figure legend, the reader is referred to the Web version of this article.)

Compared with 50% PH-only groups, the liver growth ratio was significantly lower in the wild-type mice receiving the 2-hit injury (Fig. 2A and C). The liver mass of Selp−/− mice that received 2-hit injuries were restored to levels similar to those that only received 50% PH (Fig. 2A and C). Fig. 3 shows liver function after PH that were determined by measuring serum concentrations of ALT (liver enzyme), total bilirubin (liver metabolism), and albumin (liver biosynthesis). In comparison to their 50% PH-only counterparts, Selp−/− mice that received 2-hit injuries were found to have significantly higher serum ALT and total bilirubin levels (Fig. 3A and C). However, ALT levels were significantly lower in the Selp−/− group when compared to the wild-type group (Fig. 3A), indicating that liver function post extensive hepatocyte injury was better restored in Selp−/− mice. There were no differences in serum levels of albumin among the treatment groups (Fig. 3C).

### 3.4 3-4. Attenuated hepatic pro-inflammatory reaction in P-selectin deficiency mice after 2-hit injury of HSR and 50% PH

**Fig. 4:**
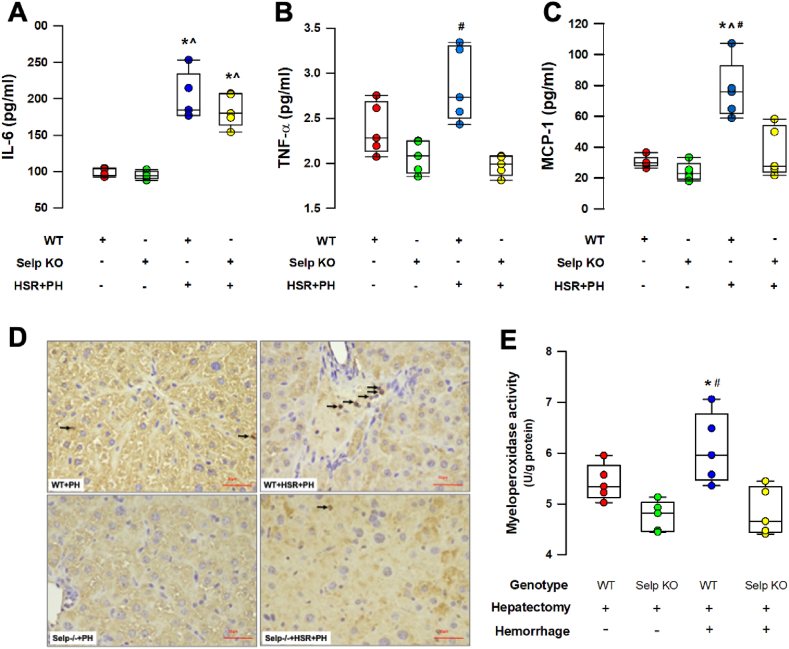
**A-C.** Tissue concentrations of pro-inflammatory cytokines in the regenerated remnant liver at 72h after 50% partial hepatectomy (PH) with or without hemorrhagic shock (HS) in wild type (WT) and P-selectin deficient (Selp −/−) mice. IL-6: interleukin-6; TNF-α: tumor necrosis factor-α; MCP-1: monocyte chemoattractant protein-1. **4D.** Representative histologic sections of the regenerated liver. Arrows indicate the expression of myeloperoxidase. **4E.** Enzymatic activity of myeloperoxidase in the liver homogenates of mice exposed to PH with or without HS. *P < 0.05 vs PH only (within groups); ^P < 0.05 vs PH only (between groups); ^#^P < 0.05 vs Selp −/− with HS + PH. n = 5 different animals in each group; data were analyzed using the Kruskal-Wallis test by rank. Results are presented as box-and-whisker plots, in which the horizontal lines of color boxes indicate the 75th percentile, median and 25th percentile of the distribution, and the upper and lower whiskers indicate the maximal and minimal values. (For interpretation of the references to color in this figure legend, the reader is referred to the Web version of this article.)

Tissue concentrations of IL-6, a potent hepatocyte mitogen during liver regeneration [13] were significantly elevated in mice receiving HSR and 50% PH (Fig. 4A). Concentrations of other pro-inflammatory cytokines (TNF-α and MCP-1) in the remnant liver increased significantly in the wild-type group but were returned to base levels in the Selp−/− mice (Fig. 4B and C). Sections of the regenerated liver showed higher levels of leukocyte infiltration (Fig. 4D) and myeloperoxidase activity (Fig. 4E) in the hepatic parenchyma of wild-type mice that received the 2-hit injury. In the regenerated livers of Selp−/− mice that received 2-hit injuries, the number of interstitial leukocytes and myeloperoxidase activity were significantly suppressed when compared to the wild-type counterparts (Fig. 4D and F).

### 3.5 3-5. Enhanced hepatocyte proliferation indices in P-selectin deficiency mice after 2-hit injury of HSR and 50% PH

**Fig. 5:**
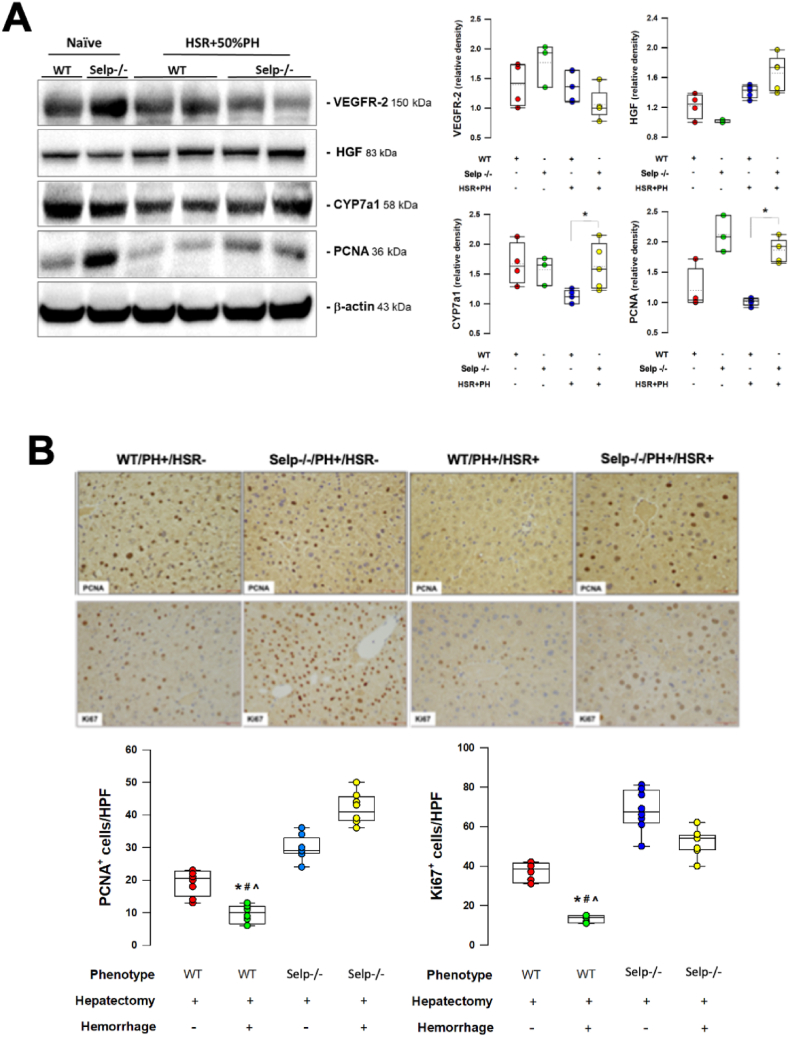
Proliferation indices of the regenerated remnant liver at 72h after 50% partial hepatectomy (PH) with or without hemorrhagic shock (HS) in wild type (WT) and P-selectin deficient (Selp −/−) mice. **A.** Western blotting analysis of the protein expressions of vascular endothelial growth factor receptor (VEGFR)-2, hepatic growth factor (HGF), cytochrome P450 7a1 (CYP7a1) and proliferating cell nuclear antigen (PCNA) in the regenerated liver homogenates. **B.** Immunohistochemical expressions of Ki67 and PCNA in the regenerated remnant liver sections. *P < 0.05 vs Selp −/− with HS + PH; ^#^P < 0.05 vs WT with PH only; ^P<0.05 vs Selp −/− with PH only. n = 5 different animals in each group; data were analyzed using the Kruskal-Wallis test by rank. Results are presented as box-and-whisker plots, in which the horizontal lines of color boxes indicate the 75th percentile, median and 25th percentile of the distribution, and the upper and lower whiskers indicate the maximal and minimal values. (For interpretation of the references to color in this figure legend, the reader is referred to the Web version of this article.)

The two-hit model of HSR and 50% PH induced the expression of HGF in the remnant livers of wild-type and Selp−/− mice (Fig. 5A), suggesting mitogenic proliferation of hepatocytes after hepatic injury. Compared with Selp−/− mice, the expressions of other hepatocyte proliferation indices (CYP7a1 and PCNA) were significantly lower in wild-type animals (Fig. 5A). The number of PCNA+ and ki67+ cells were also significantly lower in wild-type mice that received HSR and 50% PH (Fig. 5B).

## 4 Discussion

**Fig. 6:**
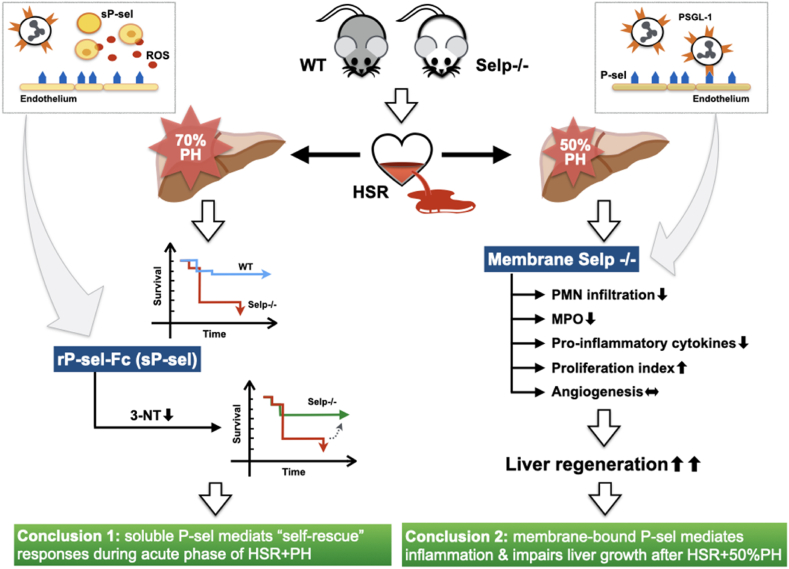
Graphical abstract of the study. Wild-type (WT) and P-selectin deficient (Selp −/−) mice were randomized assigned to receive hemorrhagic shock resuscitation (HSR) and 70% or 50% partial hepatectomy (PH). After 70% PH and HSR, the overall mortality was significantly increased in Selp −/− mice and the survival rate was improved in Selp −/− mice received pre-treated with recombinant P-sel IgG-Fc fusion protein (rP-sel-Fc), suggesting that soluble form P-selectin (sP-sel) in the systemic circulation mediates “self-rescue” responses that are essential for host survival from the acute phase of major traumatic liver injury. In the other “2-hit” model with 50% PH, Selp −/− mice were associated with enhanced liver regeneration and attenuated regional inflammatory reactions in the regenerated remnant liver, suggesting that membrane-bound P-selectin mediates infiltration of pro-inflammatory neutrophils that may impair hepatic growth during the proliferative phase of liver regeneration. MPO: myeloperoxidase; PMN: polymorphonuclear leukocytes; 3-NT: 3-nitrotyrosine; ROS: reactive oxygen species.

In this study, we characterized a “2-hit” model of HSR and PH to mimic the clinical scenario of a traumatic liver injury with hypovolemic shock that requires life-saving resection of the injured liver segments [14]. Clinically, the prognosis after resection of the complex liver injury is affected by high mortality rates during the early post-operative phase (up to 13%) and the early survival rate is also determined by the capability of the liver to regenerate following extended hepatectomy [15]. Therefore, models of HSR followed by 70% PH or 50% PH were used in this study to determine the survival outcomes or degrees of liver regeneration after “2-hit” injuries, respectively (Fig. 6).

A previous study has demonstrated that activation of P-selectin in the microvascular endothelium is essential for the adhesive interactions with leukocyte P-selectin glycoprotein ligand (PSGL)-1 and the subsequent upregulation of the tissue inflammatory response occurring in hemorrhagic shock [9]. On the other hand, P-selectin can also be secreted from activated platelets and endothelial cells into the systemic circulation as a soluble form P-selectin (sP-Sel) [16]. sP-Sel inhibits the adhesion of leukocytes onto inflamed tissue and mediates an anti-inflammation effect [17]. Chang and colleagues found that blood concentrations of sP-sel significantly increased after being challenged with venom or anthrax, and pretreatment with sP-Sel (recombinant P-sel IgG-Fc fusion protein) exerted protective effects against coagulopathy and mortality in wild-type mice [18,19]. Furthermore, P-sel neutralizing antibody injections reduced circulating proinflammatory cytokine (TNF-α and IL-1β) levels, and markedly reduced the mortality rates of venom-challenged mice [18,19]. Therefore, we believe an investigation regarding the dual effects of P-selectin (membrane bound vs soluble forms) in traumatic hemorrhagic shock and tissue injury is imperative [20].

In the first experiment, mice underwent HSR followed by an extensive liver resection (70% PH), a combination that is associated with high postoperative mortality. Compared with wild-type animals, Selp−/− mice were found to have significantly higher mortality rates at 8 h after injury. No Selp−/− mice survived after 48 h, while 60% of the wild-type mice survived up to 72 h after injury. However, when the Selp−/− mice were pretreated with rP-sel-Fc, the overall survival rate was restored to levels similar with wild-type animals after HSR and 70% PH. This supports the hypothesis that circulating sP-Sel may serve as a “self-rescue” response that is essential for host recovery from the acute phase of lethal hypocoagulation and pro-inflammatory reactions secondary to viper venom exposure or ischemia-reperfusion injuries [[18], [19], [20]]. Selp−/− mice that survived 72h post 70% PH were found to have significantly lower levels of nitrotyrosine in their remnant liver tissues. Hemorrhagic shock and extended liver resection are associated with increased systemic oxidative stress and pro-inflammatory reactions that lead to the overproduction of free radicals (including nitric oxide and superoxide anions). This process then generates an oxidizing intermediate, peroxynitrite (ONOO-) [21]. ONOO- nitrates bind tyrosine to form 3-nitrotyrosine which impairs the biological function of proteins [22].

This study further investigated whether the genetic deletion of P-selectin can affect liver regeneration after hemorrhagic shock and PH due to the lack of selectin-mediated leukocyte recruitment during early phases of the “2-hit” injury. In the second experimental protocol, animals were subjected to a similar level of hemorrhagic shock but was followed by a less extensive liver resection (50% PH). In this protocol, almost no postoperative mortalities were recorded in both the wild-type and Selp−/− mice. The aim of this second study protocol was to compare the regenerative capacities of the remnant liver in wild-type and P-selectin deficient mice after the acute phase post PH with or without preexisting HSR. Wild-type and Selp −/− mice that underwent the “2-hit” injury were found to have significantly higher serum levels of ALT and bilirubin compared to the PH-only mice. This suggests that hemorrhagic shock can provoke additional hepatic injuries due to hypoperfusion in the liver parenchyma that results in the activation of leukocytes and increased leukocyte-endothelial interactions [23]. Three days after injury, wild-type mice that received the “2-hit” injury had significantly lower body weights and liver growth ratios compared to their P-selectin deficient counterparts or mice received only 50% PH. Gross examination found tissue volumes were reduced in all five liver lobes, and the overall mass of the re-growth remnant liver was also reduced in the wild-type mice exposed to the “2-hit” injury. This suggests the decrease in liver regrowth in mice that received 50% PH is a result of impaired general regenerative capacity rather than a regional effect of the remnant liver. On the other hand, the general conditions of the Selp−/− mice, determined by body weight change after the “2-hit” injury, did not differ significantly from the PH-only groups Liver growth mass in these mice was also significantly higher than the wild-type mice that received the “2-hit” injury. Furthermore, significantly lower serum ALT levels suggest a better degree of liver function recovery in the Selp−/− animals than the wild-type group.

The degree of inflammation in the remnant liver were determined by tissue pro-inflammatory cytokines, leukocyte infiltration, and myeloperoxidase activity. MCP-1/CXCL2 is one of the key chemokines that regulate migration and infiltration of monocytes/macrophages through the capillary endothelial barrier. Suppression of MCP-1/CXCL2 has been shown to reduce regional chemotaxis and inflammatory reactions in the injured liver after ischemia-reperfusion [24]. In this study, MCP-1 and myeloperoxidase enzymatic activity increased significantly in the liver tissues of wild-type mice that received the “2-hit” injury, but these two regional pro-inflammatory markers were significantly lower in the Selp−/− counterpart group. It is generally believed that the beneficial effects of P-selectin depletion in tissue regeneration are mediated by attenuation of regional inflammation rather than providing direct effect on cell proliferation [8].

The results of this study need to be interpreted in light of the limitations below. First, the mortality rates of the experimental animals post HSR and 70% PH were extremely high and most animals in the Selp −/− group died within 48h postoperatively, which precluded fixed measuring time points for blood samples and post-operative tissue biopsies. Therefore, we were not able to provide sufficient quantitative measurements for investigating the mechanisms underlying the survival benefits seen in Selp−/− mice treated with rP-sel-Fc. Second, blunt abdominal injuries are usually associated with a certain degree of parenchymal injury in the remnant liver lobes. However, the animals in this 2-hit model only underwent partial hepatectomies without inducing parenchymal damage in the remnant liver. Therefore, the regenerative process of the remnant liver in this experimental model might not be fully applicable to traumatic liver injury recovery in a clinical context. Third, the expressions of the other two selectin members, namely L- and E-selectins were not measured in this study, as these two membrane-bound selectins might act redundantly to circumvent P-selectin depletion. However, in mice with genetically depleted of one, two, or three selectins, leukocyte homeostasis and recruitment to inflammatory sites are dominantly governed by the functions of P-selectin rather than the other two selectins [25]. Forth, plasma levels of aspartate transaminase (AST) and other liver enzymes were not analyzed in this study due to very limited blood samples were collected from each mouse. Fifth, several retrospective clinical studies found inverse relationships between plasma P-selectin levels and general outcomes in patients with hemorrhagic shock [26] or asthma [27]. However, the causal relationship between elevated plasma P-selectin levels and the disease outcomes could not be established from the results of these clinical observational studies, further controlled studies are required to define the actual biological role of the native plasma P-selectin during physiological challenges.

## 5 Conclusion

This pre-clinical model identifies two distinct phenotypic characteristics of soluble-form vs membrane-bound P-selectin gene in the critical conditions induced by hemorrhagic shock and partial hepatectomy. Soluble form circulating P-selectin mediates crucial survival benefits during the early acute stages of hemorrhagic shock and extensive liver parenchyma loss (Fig. 6). Membrane-bound P-selectin mediates regional pro-inflammatory reactions in the remnant liver after the acute stage of the “2-hit” injury, thereby impairing hepatic regeneration during the proliferative phase (Fig. 6). The results of these experimental works may provide molecular mechanistic insight and clinical therapeutic applications of P-selectin in the acute and regenerative phases of traumatic hepatic injury.

## Contribution of authors

J.L.C., H.H.C. and C.F.L. designed the study. J.L.C. and T.T.C. performed the animal experiments and recorded the in vivo measurements. C.C.H. and T.T.C. performed the protein analysis and ELISA assays. J.L.C., T.T.C. and C.C.H. performed tissue collection. J.L.C., H.H.C. and C.F.L. contributed to the statistical analysis and interpretation of data. J.L.C. and C.F.L. contributed to drafting the manuscript. All authors read and approved the final version of manuscript.

## Funding statement

Dr. Chen Fuh Lam was supported by 10.13039/501100004663Ministry of Science and Technology, Taiwan {MOST 107-2314-B-650-004}, E-Da Hospital {EDPJ110069, EDPJ107060, EDPJ108058}.

Hsin-Hou Chang was supported by Hualien Tzu Chi Hospital, 10.13039/501100005925Buddhist Tzu Chi Medical Foundation {TCMMP104-06-01 and TCMMP108-04-01}.

## Data availability statement

Data will be made available on request.

## Declaration of competing interest

The authors declare that they have no known competing financial interests or personal relationships that could have appeared to influence the work reported in this paper.
