# Supplementary material for: Dual phenotypic characteristics of P-selectin in a mouse model of hemorrhagic shock and hepatectomy
Source: Heliyon. 2023 Jul 28;9(8):e18627. doi: 10.1016/j.heliyon.2023.e18627 (PMC10404689; doi:10.1016/j.heliyon.2023.e18627)
Supplement: Multimedia component 1 [file mmc1.pdf]

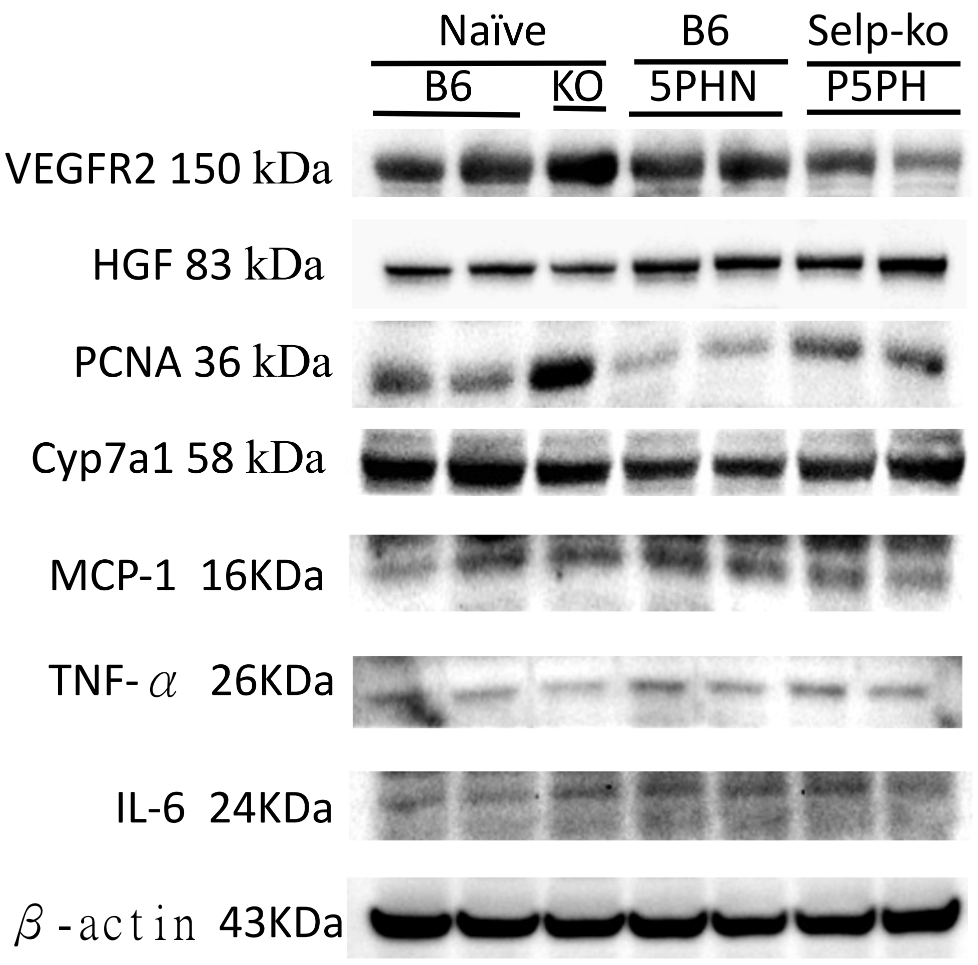

Groups:
Naïve: normal mice
B65PHN: 50%PH+HS
Selp Ko P5PH:50%PH+HS

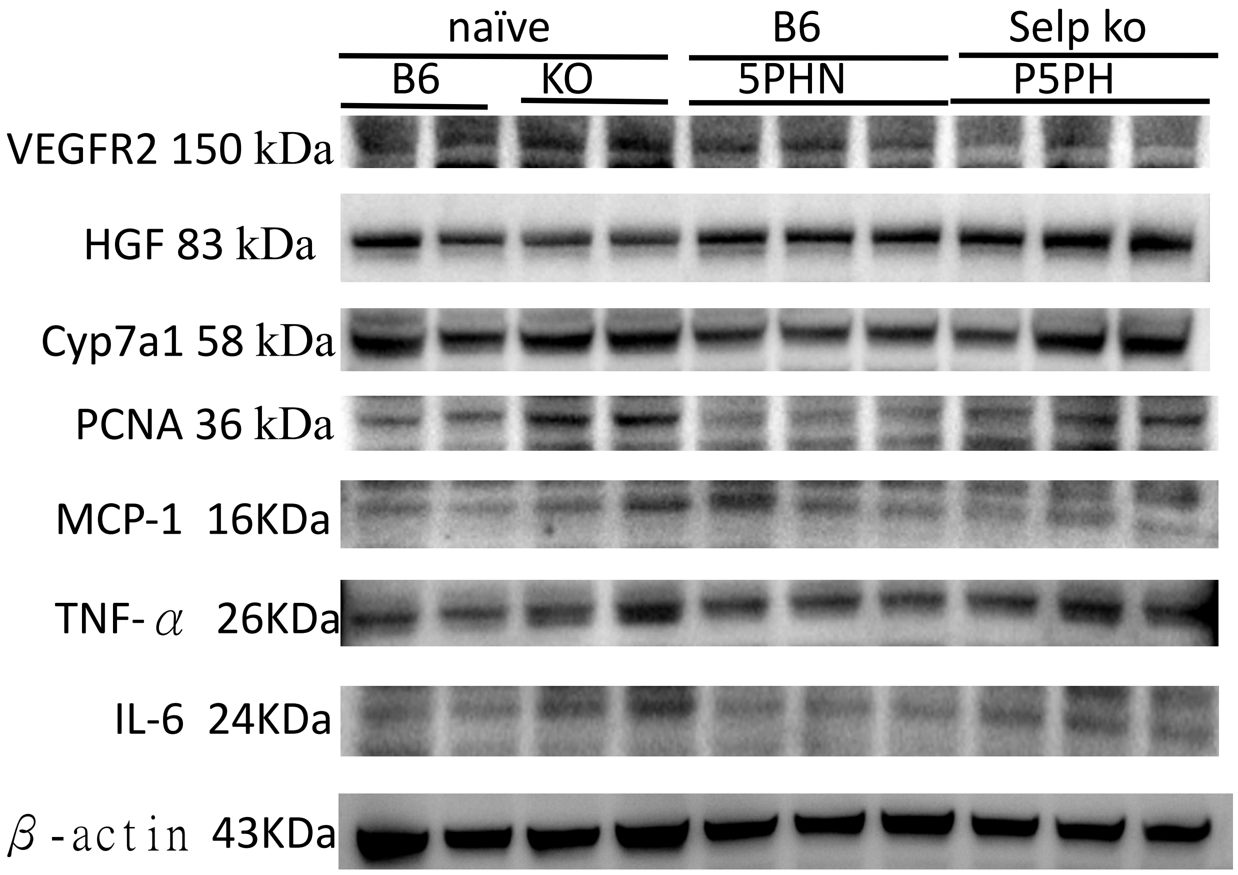

Groups:
Naïve: normal mice
B65PHN: 50%PH+HS
Selp Ko P5PH:50%PH+HS

antibodies

VEGFR2(1:500) , NOVUS(NB100-2382)

IL-6 (1:1000) , cell signaling(12912)

TNF-α (1:1000), Gene Tex(GTX110520)

HGF (1:1000), Gene Tex(GTX32651)

PCNA (1:1000) , BD Biosciences (610665)

MCP-1(1:1000) , NOVUS(NBP1-07035)

Cyp7a1(1:1000), abcam(ab65596)

β -actin (1:5000) , MILLIPORE(MAB1501)
